# Feasibility and Early and Midterm Outcomes of Midaortic Syndrome: A Retrospective Cohort Study

**DOI:** 10.3390/jcm15010036

**Published:** 2025-12-20

**Authors:** Hamad Algedaiby, Maher Fattoum, Michael Keese

**Affiliations:** 1 Department of General and Specialized Surgery, College of Medicine, Taibah University, Medina 42353, Saudi Arabia; hgedaiby@taibahu.edu.sa; 2 Department of Vascular and Endovascular Surgery, Brüderklinikum Julia Lanz Hospital, 68165 Mannheim, Germany; mmf9474@yahoo.de

**Keywords:** Midaortic Syndrome, endovascular repair, chimney technique, renal artery, visceral artery, hypertension

## Abstract

**Background**: Midaortic Syndrome (MAS) is a rare vascular condition characterized by segmental narrowing of the thoracic and abdominal aorta, often involving ostial narrowing of the renal or visceral arteries. While open surgical repair has been the standard treatment, it carries significant morbidity, especially in high-risk patients. Endovascular techniques, including the Chimney approach, provide a minimally invasive alternative to preserve and reestablish both aortic and branch vessel perfusion. This study evaluates the feasibility, safety, and early and midterm outcomes of the Chimney technique used in a cohort of patients with MAS. **Methods**: Between 2019 and 2025, 9 patients with MAS and branch vessel involvement underwent endovascular repair using the Chimney technique at Brüderklinikum Julia Lanz Hospital in the Mannheim Teaching Hospital of Heidelberg University. Pre-procedural planning was based on computed tomography angiography. Technical success, peri-procedural complications, changes in blood pressure, renal function, and target-vessel stent patency were monitored. Patients were followed over a median of 3 years (range, 0.08–6 years). **Results**: Nine patients (mean age 77.2 ± 8.7 years; 66.6% female) underwent endovascular repair for midaortic syndrome. All patients were unfit for open surgery. Comorbidities included hypertension (100%), coronary artery disease (100%), and chronic kidney disease (77.7%). Technical success and target-vessel patency were 100%, with no intraoperative deaths, impairment of renal function, or 30-day mortality. One patient (11.1%) developed an access-site hematoma, which was managed conservatively. Median hospital stay was 6 days. During a median 3-year follow-up (range 1 month–6 years), all chimney stents remained patent, patients experienced durable symptom relief, blood pressure improvement, and freedom from reintervention. **Conclusions**: The Chimney technique offers a safe and effective endovascular option for high-risk patients with Midaortic Syndrome, achieving high technical success, preserved branch-vessel patency, and improvement of symptoms. Larger studies with longer follow-up are warranted to confirm durability and optimize patient selection for this technique.

## 1. Introduction

Midaortic Syndrome (MAS) is a rare vascular disorder characterized by segmental narrowing of the distal thoracic or abdominal aorta, often accompanied by ostial stenosis of the renal and visceral arteries. The condition was initially reported by Sen and colleagues in 1963 [1]. It typically affects younger individuals and manifests with severe hypertension, claudication, or end-organ dysfunction. MAS is a markedly uncommon vascular disorder, representing less than 1% of aortic coarctation cases and an estimated incidence of roughly 1 per million individuals [2,3,4]. In untreated patients, mortality rates may reach up to 90% by the sixth decade of life due to progressive end-organ damage [3].

The etiology of MAS is heterogeneous and has traditionally been classified as idiopathic, congenital, or acquired. Congenital forms are often related to abnormal embryologic development of the aorta and typically manifest in childhood or adolescence. Acquired cases may result from inflammatory vasculopathies such as Takayasu arteritis or giant cell arteritis, as well as fibromuscular dysplasia or atherosclerosis in adults [2,3]. More recently, advances in genetic testing have revealed that many cases previously labeled as idiopathic or acquired can be attributed to underlying genetic mutations [4,5]. Regardless of the cause, the narrowing of the aorta and its branch vessels produces significant hemodynamic compromise and represents a therapeutic challenge. Owing to its progressive course and potential for significant morbidity, timely diagnosis and effective intervention are essential.

Historically, open surgical repair has been considered the standard of care for MAS. However, it carries considerable perioperative risks, especially in patients with extensive disease or multiple comorbidities. Over the past few decades, Thoracoabdominal endarterectomy (TEA) and visceral bypass grafting have been established surgical strategies for managing complex renovisceral or middle aortic lesions. TEA involves longitudinal exposure of the thoracoabdominal aorta with removal of obstructive intimal and medial lesions, thereby restoring lumen patency without the need for prosthetic replacement. In contrast, visceral bypass grafting entails revascularization of the renal and mesenteric arteries using prosthetic or autologous vein conduits, typically originating from the iliac or distal aorta, to restore flow in cases where direct endarterectomy or stenting is not feasible. Both techniques have demonstrated durable long-term patency but are associated with substantial perioperative risk, limiting their application to carefully selected patients [6,7].

Despite these advantages, endovascular management of MAS remains technically demanding, especially when the stenotic segment involves major visceral or renal branch vessels.

The chimney technique, deploying branch stents in parallel to an aortic endograft to preserve target-vessel perfusion, has been widely adopted for complex juxtarenal and arch aneurysms and is now increasingly applied in the repair of thoracoabdominal aneurysms [8]. While its use in abdominal aortic aneurysms and aortic arch lesions is increasingly reported [9], multicenter and single-center chimney series report a high level of technical success (>95%). Encouraging target-vessel patency at midterm follow-up with acceptable rates of major adverse events when careful patient selection and meticulous technique are used. However, type Ia endoleak (“gutter endoleak”) and late reintervention remain recognized limitations of the technique in larger series of aneurysm patients [10].

Published reports specifically addressing endovascular management of MAS remain limited to case reports involving single or a few patients, but they consistently demonstrate feasibility, symptomatic improvement, and preserved branch-vessel patency in the short- to mid-term. Chimney-assisted strategies appear to achieve durable branch patency and clinical benefit in patients who are poor surgical candidates. There is a need for reports that include vigilant long-term imaging surveillance and the possibility of late device-related complications [11]. More recent long-term analyses of chimney and parallel graft techniques in aneurysm support durable outcomes in experienced centers: multicenter mid-term studies and single-center 7-year follow-ups report sustained patency and acceptable late survival, although some cohorts required reinterventions for late type Ia endoleaks or graft-related issues [12]. A Type Ia endoleak results from an inadequate proximal seal, whereas a gutter-related Type Ia endoleak is a distinct variant seen in chimney or snorkel EVAR, arising from peri-graft channels (‘gutters’) formed between the parallel stents and the main endograft.

These data highlight both the promise and the limitations of chimney-assisted endovascular repair and point to the need for further MAS-specific outcome data comparing techniques and patient subgroups (e.g., juvenile vs. adult presentations).

Herein, we report on challenging adult cases of MAS, all presenting with lower extremity claudication. All patients were ASA III, which rendered them unsuitable for open surgical treatment. We describe the surgical technique, preoperative and postoperative outcomes, and follow-up. Additionally, we provide a systematic review of the existing literature on MAS and endovascular chimney strategies, highlighting their emerging role as a durable, minimally invasive option in selected patients.

## 2. Materials and Methods

### 2.1. Study Design and Patient Selection

We here present a retrospective, single-center study including nine patients diagnosed with Midaortic Syndrome (MAS) who underwent endovascular repair using the Chimney technique between 2019 and 2025 at Brüderklinikum Julia Lanz Hospital, Mannheim, a teaching hospital of Heidelberg University. All nine MAS patients treated in our clinic during the study period were included, except two patients with malignant tumors and an oncologic prognosis of <6 months. These excluded patients were managed conservatively. All patients presented with severe hypertension and lower extremity claudication. Due to their significant comorbidities, including coronary artery disease, they were all grouped in ASA class III. Written informed consent was obtained from patients, and this study was approved by the institutional review board of the Brüderklinikum Julia Lanz Hospital (Approval No. F-2017-044).

### 2.2. Preoperative Evaluation

All patients underwent detailed clinical examination and pre-procedural imaging with computed tomography angiography (CTA) to evaluate the extent and distribution of aortic and branch vessel stenosis. Lesions involving the abdominal or thoracic aorta, renal arteries, superior mesenteric artery (SMA), celiac trunk, and iliac arteries were identified. Stenotic segments were defined as ≥70% luminal narrowing in all cases. Stenosis exceeding 90% was defined as narrowing of the arterial lumen by more than 90%. Preoperative assessment was based on available clinical and imaging data. Relevant patient information included a history of coronary artery disease (KHK), chronic renal failure, peripheral arterial disease (stages 3–5), and the presence of preoperative congestive heart failure.

### 2.3. Endovascular Procedure

For all nine patients, blood pressure and heart rate were strictly controlled after admission (target blood pressure 130/80 mmHg, heart rate ≤100 beats/min). All procedures were performed in a hybrid operating room under fluoroscopic guidance using an Artis 2 hybrid theater. General anesthesia with endotracheal intubation was administered in all cases.

**Figure 1:**
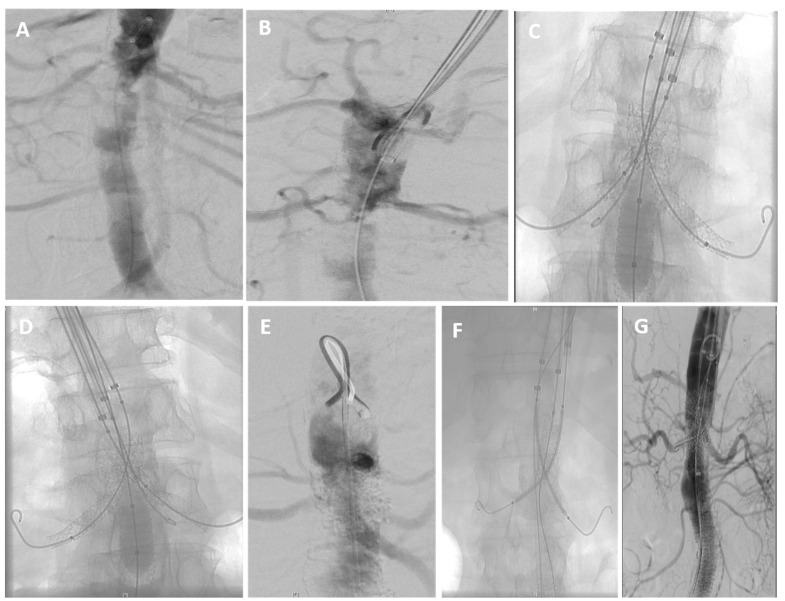
(**A**–**G**) Intraoperative angiogram demonstrating chimney stent placement for endovascular repair of midaortic syndrome. The image shows the deployment of a parallel stent extending into the branch vessel alongside the main aortic endograft, which maintains perfusion while addressing the aortic narrowing.

Vascular access was achieved either by surgical cutdown or percutaneous puncture, depending on patient anatomy and lesion characteristics. Access sites included the femoral, subclavian, and axillary arteries. For the aortic intervention, covered stents Advanta V12 (Getinge, Gothenburg, Sweden) and BeGraft Plus (Bentley, Hechingen, Germany) were deployed across the diseased aortic segment. In patients with distal extension, additional stent grafts were overlapped to ensure complete coverage. In all cases, the superior mesenteric and/or renal arteries were involved. Parallel balloon-expandable stents BeGraft Plus (Bentley, Hechingen, Germany) and Smart (Cordis, FL, USA) were advanced over Rosen wire (Cook Medical, IN, USA) and deployed simultaneously with the aortic stent using the Chimney technique. Concomitant iliac artery stenoses were treated with balloon-expandable or self-expanding nitinol stents. In patients with bilateral common femoral occlusion, open thromboendarterectomy was performed in 3 patients. All stents were precisely positioned under fluoroscopic guidance, and high-pressure balloon post-dilatation was performed as needed to optimize stent expansion. Operative images of the chimney stent placement were utilized to depict the procedural technique (Figure 1).

### 2.4. Postoperative Management and Follow-Up

Patients were kept in the intensive care unit for 24–48 h to monitor end-organ function. Dual antiplatelet therapy (aspirin and clopidogrel) was initiated postoperatively and continued for 6 months. Statin was started preoperatively. Duplex sonography and CTA once after 6 months and then yearly, including blood pressure monitoring and serum creatinine assessment.

### 2.5. Outcome Measures

The primary outcomes were technical success, defined as restoration of vessel patency to more than 70% following the procedure, peri-procedural complications, and 30-day mortality. Secondary outcomes included improvement in blood pressure control, renal function changes, target-vessel patency, referred to primary patency, meaning that the treated vessels remained open without evidence of restenosis and without requiring repeat revascularization during follow-up, and long-term survival.

### 2.6. Statistical Analysis

Descriptive statistics were used to summarize patient demographics, procedural details, and outcomes. Continuous variables are presented as mean ± standard deviation (SD) or median with range as appropriate. Categorical variables are expressed as counts and percentages. Survival analysis was performed using the Kaplan–Meier method to estimate overall survival over follow-up. Patients without events (death) were censored at the last follow-up. Given the small sample size (*n* = 9), no formal comparative statistics were performed.

## 3. Results

### Patient Characteristics

In total, nine patients with midaortic syndrome (MAS) were treated using the Chimney technique between 2019 and 2025.

The mean age was 77.2 years (range, 68–89 years), with a predominance of females (*n* = 6) compared to males (*n* = 3). All patients presented with long-standing hypertension that was refractory to multi-drug therapy. Comorbidities included diabetes mellitus (*n* = 4; 44.4%) and active smoking (*n* = 5; 55.5%). Patient characteristics are shown in Table 1A. All patients had coronary artery disease, and all were classified as American Society of Anesthesiologists (ASA) class III due to their overall risk profile, rendering them unsuitable candidates for conventional open surgical repair. The procedural and postoperative care of the nine patients undergoing Chimney–TEA procedures is summarized in Table 1B.

**Figure 2:**
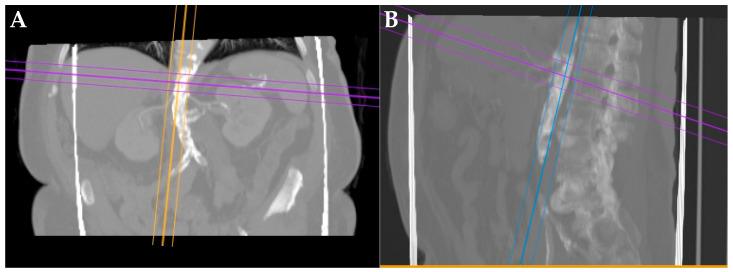
(**A**) Preoperative coronal and (**B**) sagittal computed tomography angiography (CTA) images showing severe narrowing of the abdominal aorta consistent with midaortic syndrome. Multiplanar reconstruction lines indicate the planes selected for further evaluation and planning of endovascular chimney repair.

These findings align with the reported propensity for MAS to present with renovascular hypertension and complex comorbid profiles in adults. Preoperative imaging (Figure 2A,B) with computed tomography angiography (CTA) demonstrated diffuse narrowing of the thoracoabdominal aorta in all cases, with stenosis ranging from 70% to 90%. Bilateral renal artery stenosis was observed in five patients, while unilateral stenosis was present in the remaining three.

Endovascular repair was successfully performed in all nine patients using balloon-expandable covered stents with the Chimney technique employed to preserve visceral and renal artery perfusion. Technical success was achieved in 100% of cases. The mean procedural duration was 73.4 min (range: 55–92 min), with an average fluoroscopy time of 19.8 min. Importantly, no patients experienced intraoperative or postoperative acute renal failure or ischemic complications, as shown in Table 1B.

**Figure 3:**
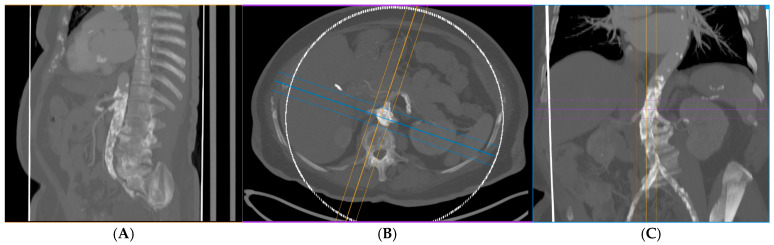
(**A**) Postoperative computed tomography angiography (CTA) images in sagittal, (**B**) axial, and (**C**) coronal planes following endovascular chimney repair for midaortic syndrome. The images demonstrate well-positioned stent grafts with satisfactory patency of the aortic lumen and visceral branch vessels.

During a median follow-up of 3 years (range 1 month–6 years), all stents and target vessels remained patent (100% target-vessel patency) with no cases of restenosis or target-lesion revascularization. Follow-up duplex sonography and CT angiography at 5 years confirmed sustained patency of the chimney stent grafts, as shown in Figure 3A–C. No patients developed renal impairment during follow-up, and importantly, no mortality occurred in this cohort over the observation period. Postoperative outcome is summarized in Table 2. The results demonstrated 100% overall survival, with no cases of reintervention or patency loss throughout the follow-up period. All patients remained free from amputation. There was a significant improvement in walking distance, and all patients reported relief from claudication symptoms. Additionally, each patient showed improvement of at least one stage according to the Fontaine Classification.

Collectively, these findings reinforce that endovascular repair with chimney/parallel graft strategies can achieve durable stent and target-vessel patency with favorable outcomes, although lifelong surveillance remains essential.

## 4. Discussion

Midaortic syndrome (MAS) remains a challenging clinical entity owing to its complex anatomical involvement and the need for durable revascularization. Open surgical repair is still considered the gold standard [13] due to its definitive anatomic reconstruction and proven long-term durability. However, these procedures are technically demanding and carry substantial perioperative morbidity and mortality, particularly in younger patients with diffuse disease and in older individuals with comorbidities. Over the past two decades, the evolution of endovascular techniques has provided a less invasive alternative, with the potential to achieve durable revascularization while reducing procedural risk in MAS patients [14].

In our series of nine patients with MAS, endovascular repair using the chimney technique achieved 100% technical success, with restoration of renal and visceral perfusion. No perioperative mortality or renal impairment occurred, and all patients demonstrated improvement in blood pressure control, with reduced dependence on antihypertensive medications. Importantly, target-vessel patency remained 100% during a median follow-up of 3 years, and no secondary interventions were required. These findings highlight the safety, efficacy, and reproducibility of the chimney technique in this challenging patient population. These favorable outcomes are consistent with broader multicenter experiences demonstrating that the chimney technique provides effective preservation of branch vessel perfusion while minimizing the perioperative morbidity associated with open surgical bypass [15].

Previous studies further support these outcomes. Bin et al. (2013) [16] reported durable patency and improved renal function following chimney-assisted repair of juxtarenal aortic occlusion, with follow-up extending up to six years. The study included ten high-risk female patients (mean age, 68 years), of whom seven had renal artery stenosis. The procedure was associated with a 10% mortality rate and an average hospital stay of two weeks.

Similarly, Hogendoorn et al. (2013) [17] demonstrated 100% chimney stent patency over a median follow-up of 11 months, while mid- and long-term series have shown primary patency rates exceeding 93% at 36–48 months [18]. Results from the PERICLES registry also confirmed durable branch-vessel patency (~92%) at mid-term follow-up in complex aortic pathologies [19]. A systematic review noted that in adult MAS populations, renal artery stenosis occurs in about 63% of cases and visceral artery involvement in about 33%, with refractory hypertension being the leading indication for intervention [3]. While previous studies by Patel et al. 2020 [20] have characterized the long-term outcomes of mixed open and endovascular approaches in Midaortic Syndrome, and Liu et al. 2024 [21] have demonstrated durability of endovascular repair in adult patients (with 5- to 10-year reintervention-free survival rates ~92.3% and ~79.1%), there remains a paucity of data specifically evaluating parallel graft (chimney) techniques in MAS, particularly in older, high-risk populations unfit for open surgery. Our series is novel in demonstrating that, in a comorbid, elderly cohort (mean age ~77 years), the chimney technique can safely achieve 100% technical success, preserve renal/visceral perfusion, provide durable symptom relief, and achieve freedom from reintervention over a median 3-year follow-up. Importantly, we observed no type Ia gutter endoleaks (no AAA patients involved) and no renal impairment or 30-day mortality, outcomes which compare favorably to the published experiences of parallel grafts in aneurysmal contexts but in a more challenging non-aneurysmal disease. This suggests that in carefully selected MAS patients unamenable to open repair, chimney endovascular reconstruction may offer a minimally invasive, durable, and effective alternative. Nonetheless, given the small sample size and limited follow-up, larger prospective or registry-based studies are needed to validate these findings and define patient/lesion characteristics most predictive of success. Table 3A,B provides a comparative overview of the present series with key published experiences of endovascular and chimney graft repair for midaortic syndrome (Table 3A) and juxtarenal aortic disease (Table 3B).

The chimney technique, initially developed for juxtarenal and thoracoabdominal aneurysms, has since been adapted to other anatomically challenging scenarios, including MAS. A systematic review of chimney grafts for visceral vessel preservation reported a technical success rate close to 100% and chimney graft patency of approximately 98% during mid-term follow-up [28]. Similarly, in thoracic endovascular aortic repair (TEVAR) for arch pathologies, Li et al. (2018) [29] observed technical success in 91% of cases, with high rates of branch preservation. These findings parallel our results, where 100% target-vessel patency and absence of procedure-related complications were achieved in a non-aneurysmal MAS cohort, further supporting the adaptability and effectiveness of chimney techniques in complex off-label settings.

Across aneurysmal disease, outcomes with parallel-graft techniques have been encouraging. Kopp et al. (2025) [30] reported 90% secondary technical success and 94% 3-year patency in complex ruptured aneurysms, though with higher early gutter endoleaks when multiple grafts were used, while Prapassaro et al. (2022) [31] and Takeuchi et al. (2024) [32] similarly demonstrated good mid-term patency and survival with ChEVAR in JAAA and RAAA. In contrast, our MAS cohort achieved 100% target-vessel patency with no gutter endoleaks or reinterventions, likely reflecting the more favorable hemodynamics of non-aneurysmal aortic pathology and the robust performance of the chimney technique in this setting.

Despite these encouraging outcomes, chimney repair is not without challenges. Endoleaks, particularly gutter-related type Ia endoleaks, are a recognized complication of parallel grafting, with a pooled early type Ia endoleak rate of approximately 9% (95% CI: 6.5–13.4%) based on systematic review data [33]. As here we only report on MAS patients, none were observed in our cohort. In aneurysm patients, meticulous pre-procedural planning, precise stent sizing, and high-quality intraoperative imaging are essential to minimize this risk. Furthermore, while our follow-up revealed sustained patency and improved blood pressure control, long-term durability of chimney repair in MAS remains uncertain, particularly in younger patients. Vigilant surveillance and readiness for secondary interventions thus remain critical to ensure sustained success.

### Limitations

This single-center study is limited by its retrospective design, small sample size, and lack of a control group. Although all procedures were performed by an experienced team in a high-volume center, the findings may not be generalizable.

## 5. Conclusions

The endovascular repair using the chimney technique seems to be a safe, effective, and durable alternative to open surgery for MAS. The combination of 100% technical success, preserved renal and visceral perfusion, absence of procedure-related renal impairment, and 100% target-vessel patency observed in this cohort highlights its promise as a first-line treatment option in carefully selected patients. However, these findings should be interpreted with caution, given the retrospective, single-center design and small sample size. Larger multicenter studies with extended follow-up are warranted to confirm these outcomes, refine patient selection, and optimize technical strategies aimed at reducing complications and ensuring long-term durability.

## Figures and Tables

**Table 1:** (**A**) Clinical and Demographic Profile of Patients Undergoing Chimney–TEA procedures (*n* = 9). (**B**) Procedural Details, Complications, and Postoperative Care in Patients Undergoing Chimney–TEA.

**(A)**	
**Variable**	**Values**
Sex (M/F)	3/6
Mean age (years)	77.2 (range 68–89)
History of the ICA stent	3/9 (33.3%)
ASA class	III in all patients
Smoking history	5/9 (55.5%)
Comorbidities	CAD: 9/9 (100%); COPD: 1(11.11); Chronic renal failure: 7/9 (77.7%); HTN: 9/9 (100%); PAD: 9/9 (100%); CHF: 7/9 (77.7%); Diabetes mellitus: 4/9 (44.4%)
**(B)**	
**Variable**	**Values**
Chimney procedure performed	9 (100%)
With Femoral endarterectomy	3/9 (33.3%)
Preoperative Angiography	6/9 (66.6%)
Mean operative time	73.4 min (range 55–92)
Mean radiation time	19.8 min (range 12–30)
Access route:	
1-Percutan (Femoral and Transaxillary)	5/9 (55.5%);
2-Percutan (only Femoral)	3/9 (33.3%);
3-Open Surgical (Femoral and Transaxillary)	5/9 (55.5%)
Wound infection	0/9 (0%)
Length of hospital stay	Median 6 days (range 4–34)
Renal function improvement (> 30% GFR)	4/9 (44.4%)
Complications	Pulmonary: 1; Cardiac: 0; Bleeding: 1; Gastrointestinal: 0
Post-op medication	Dual antiplatelets: 9/9 (100%); Antihyperlipidemics: 9/9 (100%)
Ischemic complications	Spinal cord ischemia: 0; Bowel ischemia: 0
Follow-up	Median 3 years (range 1 month–6 years)

**Table 2:** Follow-up outcomes after Chimney–TEVAR in MAS patients (*n* = 9).

Outcome	Result
Median follow-up (Years)	3 years (range 1 month–6 years)
Target vessel patency	100%
Renal impairment	None
Blood pressure control	Improved in all patients
Survival at follow-up	100%
30-day reintervention	0/9 (0%)
30-day mortality	0/9 (0%)

**Table 3:** (**A**) Comparison of the present series with key published reports on endovascular or chimney repair in midaortic syndrome (MAS). (**B**) Key published reports on endovascular or chimney repair in juxtarenal aortic disease.

**(A)**			
**Study/Year**	**Patient Cohort**	**Technique**	**Target-Vessel/Branch Patency/Freedom from Loss of Patency/Reintervention-Free Survival**
Present study (*n* = 9)	*n* = 9; MAS with renal/visceral involvement	Chimney	100% Target-Vessel Patency
Musajee et al. (2022) [22]	NA, Pediatric MAS	Open, endovascular (balloon/stent)	freedom from reintervention ~ 72% at 10 years
Patel et al. (2020) [20]	*n* = 13; Adult MAS	Open and endovascular	emphasizes multiple interventions and heterogeneity
Liu et al. (2024) [21]	*n* = 41; Adult MAS	Open and endovascular	Reintervention-free survival: open 87.7% at 5 yr, 71.7% at 10 yr; endovascular 92.3% at 5 yr, 79.1% at 10 yr
**(B)**			
**Study/Year**	**Patient Cohort**	**Technique**	**Target-Vessel/Branch Patency/Freedom from Loss of Patency/Reintervention-Free Survival**
Bin Jabr et al. (2013) [16]	*n* =10; juxtarenal aortic stenosis/occlusion	Chimney	100% Target-Vessel Patency up to 6 years in survivors.
Hogendoorn et al. (2013) [17]	*n* = 94; complex aortic disease,	TEVAR with chimney grafts	100% Vessel Patency at median follow-up (11 months).
Donas et al. (2015) [15]	*n* = 517; complex aortic pathologies	Snorkel/chimney (ch-EVAR)	Primary patency 94% at 17.1 months (secondary patency 95.3%).
Taneva et al. (2021) [19]	*n* = 517; branches; complex AAA	chEVAR	92% chimney branch patency
Li et al. (2022) [23]	*n* = 345; arch pathologies	Chimney-TEVAR	Primary and assisted chimney patency ~97%
Luo et al. (2023) [24]	*n* = 32; aortic arch repair	Chimney	100% branch patency at mid-term
Verlato et al. (2024) [12]	*n* = 51; complex AAA (juxta/pararenal)	chEVAR (parallel grafts)	Freedom from type Ia endoleak 91.8% at 7 years
Indriani (2021) [25]	One Patient	Endovascular covered stent or hybrid	Successful short-term improvement in BP and symptoms after stenting; adds to the evidence base for endovascular options in select adults.
Igari et al. (2016) [26]	*n* = 12	Ch-EVAR	Technical success 91.6%; the target vessel patency rate was 93.3%
Zlatanovic et al. (2022) [27]	Several studies pooled	chEVAR vs FEVAR vs open	Medium-term outcomes for chEVAR are comparable when FEVAR is unavailable; endoleak and reintervention rates are higher in some analyses.

## Data Availability

The datasets generated and analyzed during the current study are available from the corresponding author upon reasonable request, subject to privacy and confidentiality restrictions.

## Abbreviations

The following abbreviations are used in this manuscript:

MAS	Midaortic Syndrome
TEA	Thoracoabdominal endarterectomy
CTA	Computed tomography angiography
SMA	Superior mesenteric artery
SD	Standard deviation
TEVAR	Thoracic endovascular aortic repair
FEVAR	Fenestrated endovascular repair
ChEVAR	Chimney endovascular repair
AAA	Abdominal aortic aneurysms
